# The National Insurance Bill

**Published:** 1911-07-22

**Authors:** 


### THE NATIONAL INSURANCE BILL.

### MEETING CONVENED BY THE CHARITY ORGANISATION SOCIETY.

A largely attended meeting on this question was held on Monday, July 17, at Denison House, Victoria, S.W., under the presidency of Lord Sanderson, G.C.B. Supporting him were the Right Hon. Alfred Lyttelton, M.P., K.C., P.C., and C. S. Loch, Secretary of the Society.

The Secretary announced that he had received numerous letters from secretaries of organisations which were affected by the Bill. The Charity Organisation Society had been in communication with six hundred institutions, which had given returns, and those institutions had 12,700 employees, who were affected by the Bill, and there were 4,450 beneficiaries referred to, i.e. persons employed for their own good. Doubtless those figures could be greatly increased if there had been a little more time in which to get information. Sir Henry Burdett wrote that he was unable to attend, as he had to be present at a meeting of the King's Fund that afternoon. He thought the Society was abundantly justified in making the statement in clause 1 of its printed letter. He could vouch for that from knowledge based upon audited figures. He had been studying the whole question, including the source of revenue which must be affected, and came to the conclusion that about half the present income of many important hospitals derived from voluntary sources might be lost. He thought that the insured persons who required hospital care must at least be equal to half of the whole, and they ought to be paid pro rata by the State. That would preserve the principle of voluntary support, whereas a grant might destroy it. Sir Henry added that he had held aloof, so far, from public agitation, wishing to secure co-operative action which would help the Bill and help the hospitals.

The Chairman said he had only a few words to say in introducing the discussion. Judging from the meeting in that house a fortnight ago, many of those present regarded the National Insurance Bill with considerable apprehension, and would have preferred a measure less ambitious, less indiscriminate, and more carefully adapted to the particular class it was most intended to assist. But the Bill had been accepted in principle by the House of Commons, and if it was to become law it should be freed, as far as possible, from any palpable and remedial defects. Amongst those, not the least was that the Bill threatened to affect adversely various charitable institutions which no one could wish to see injured, such as hospitals, dispensaries, and homes for the maintenance and employment of people, who, without some assistance of that kind, could not maintain themselves. The meeting was called to consider how far those institutions were endangered by the Bill, and what amendments could be usefully advocated for the purpose of protecting them.

The Right Hon. A. Lyttelton, K.C., M.P., P.C., said he had for some time regarded such a Bill, in some form, as inevitable. When the Workmen's Compensation Acts were passed, the first ones affected only dangerous trades, and the tax which was laid on the industry for the purpose of compensating those who met with accidents in the course of their work fell upon the employer only. Then gradually the trades were widened, and now in a vast number of industries accidents to the employee were compensated by the industry through a tax upon the employer. It became obvious to those who thought the matter out that sickness, which in many cases was very subtly distinguished from accidents, would have to be compensated by some one. He was in general sympathy with the Bill, in that compensation for accidents or sickness was to be paid for not by a tax simply upon the employer, as in the case of the other Acts he was referring to, but should be insured against by a fund which should be contributed to by the employer, workman, and general taxpayer. When that principle was put into a Bill it was obvious it must encounter the passive opposition of the well-organised great charities of the kingdom. A charity should make no profit; homes made no profit out of the philanthropic work they did, but they did build up by their sacrifice, their service, and their benefactions many lives which, but for those services, would be hopeless. They had turned out boys and girls who, but for the services of those institutions, would have been supported by the State. It meant that people, voluntarily and by the sacrifice of time and money, converted into good citizens men and women who but for that service would be occupants of workhouses, or lunatic asylums, or gaols. In that way a considerable burden of cost was lifted from the shoulders of the State and placed upon the shoulders of the private individual, who, by his money and personal service, did that good work. It was almost unthinkable that the State should, seeing that it was the debtor in the transaction, appear as their creditor. In a balance sheet the State would be found to be owing those societies a considerable amount of money for having prevented those poor people from becoming a burden on the State. The position was that the State said, "We owe you a great deal, but notwithstanding that we ask you to pay both for yourself, as an employer, and for the inmates of your home as employees." He did not think it could remain in that state upon the Statute Book. With regard to the hospitals, if one was to judge from the agitation of the medical profession, a very heavy burden would be imposed upon the shoulders of that profession for a remuneration which was not extravagant. The probable effect was that if a doctor were saddled with more cases than he could attend to with leisure or comfort he would send more of his patients to the hospitals. That would increase the work of hospitals, and yet the means of dealing with the extra work by those hospitals would be diminished in regard to subscriptions. He was hoping that the present over-lapping might have been mitigated in a large well-thought-out scheme. There would be, under the Bill, more overlapping than at present, and there would tend to be a greater specialisation of work in the great organisation of healing. Dispensaries would deal with trifling cases; those which were somewhat more acute and serious would be dealt with by the practitioners, and the serious and difficult cases, which required great skill and the resources of the profession, would have to go to the hospital. But the classification must not be too sharp, and emergency cases must be freely dealt with. He did not think the Bill could take shape before the end of August, and, therefore, he had pleasure in moving the first resolution, which formed a very good modus vivendi for a time. The resolution ran: "That it is desirable that an amendment be introduced into the National Insurance Bill providing that hospitals and dispensaries and other charitable institutions or societies by which services are rendered to the assured should receive the actual cost of the expenditure they have incurred for such services, under conditions that will preserve the independence of the voluntary charitable institutions concerned." His chief desire in doing so was to safeguard the zeal, energy, and cheerfulness with which voluntary assistance, in comparison with all other assistance, did the great work of charity with which it was entrusted.

Mr. Morris (Secretary, London Hospital) said those connected with hospitals felt somewhat hurt that in a matter of that kind, in which the sick poor were concerned, the officials of hospitals were not taken somewhat into consultation. Having been at the work for two hundred years in London, the hospitals should know something about the subject. The present Bill would affect hospitals, from a financial point of view, disastrously. The expenditure would be affected, because at the London Hospital they would have to pay in premiums £850 to £900 for the insurance of their own employees, notwithstanding the fact that when an employee was ill he was treated in the hospital as a patient, and his full salary continued during that time. But the difference would be felt most on the income side. £85,000 a year at the London Hospital came in annually from voluntary help—legacies, donations, subscriptions, or grants from the three great sources, the King's Fund, the Saturday Fund, and the Sunday Fund. As the Bill stood, there was no inducement for a man to leave his money to do work which the State was going to do. £4,000 a year from one particular fund came from a penny a week collection from workmen, and there seemed no reason why a man should continue to pay 4d. a week under this scheme in addition. Much more than the finances, the work of the hospital would be affected adversely. Next year one would have the right to say to many of the patients, "You have insured yourselves, somebody has guaranteed to give you medical treatment when you are ill; you should go to the person who has guaranteed to give that treatment." But the State could not deliver the goods. Most of the sickness could not be treated in the homes of the sick poor, and many chronic cases would require long treatment, such as the paralytic, the epileptic, chronic heart cases, cancer cases, and those requiring bacteriological investigation. The Chancellor of the Exchequer said he did not want to interfere with hospital work, but when he interfered with their finances he was doing so. A hospital was not merely a place where sick people lay in bed and got well; it was the centre of laboratories where research was carried on, that sick people who never entered its walls could have better health. Whatever might be decided, those who knew most of hospitals would beg that they should not be nationalised. The spirit of the volunteer ran through the whole subject. It was that spirit which prevented the physicians and surgeons from looking upon patients as merely cases; there was a high code of honour which consulted the patient's wishes before anything was undertaken. He concluded by seconding the resolution.

Mr. William West (St. George's) supported the resolution, because the income of voluntary hospitals would be seriously affected should the Insurance Bill pass in its present form. The hospitals would then be called upon to pay £20,000 to £30,000 a year for the insurance of their employees, who would receive no medical benefit under the scheme, as they were treated in hospitals. He agreed it would be a national calamity if the voluntary system were done away with; it was the only one which stimulated all sections of the community in their work for hospitals.

Mr. Thies (Secretary, Royal Free Hospital) further supported the resolution, and put in a plea specially for the provincial hospitals, which were largely supported by working-men. In Manchester, Edinburgh, Glasgow, Bradford, and Birmingham the question was agitating administrators of hospitals even more keenly than those in London. In many respects the Bill was regarded as impossible in its present form. Mr. Lloyd George was not the man to do a foolish thing, and he therefore had hopes that Clauses 8 and 15 would be modified, and the voluntary hospitals so treated in a statesmanlike way.

Sir William Chance believed that the resolutions would be passed unanimously. He spoke on behalf of the National Association for the Feeble-minded, of which he was Chairman. That Association was dealing with six hundred to seven hundred people of the feeble-minded class. Those homes were struggling to keep themselves in existence, and the Bill would make it impossible to carry them on. The principle of the Bill was very small compared with its details, and it was most important that the measure should not be rushed through Parliament, considering the enormous interest at stake.

Mr. Percy Gates, as Chairman of a Provident Dispensary, supported the resolution, and said the Bill would be a great disaster to the work of such institutions. He had written to Mr. Lloyd George on the subject, but the reply he received was merely enigmatical. Any Bill which depreciated the independence of the poor should not be encouraged.

The resolution was carried unanimously.

